# Soil Water Sensor Performance and Corrections with Multiple Installation Orientations and Depths under Three Agricultural Irrigation Treatments

**DOI:** 10.3390/s19132872

**Published:** 2019-06-28

**Authors:** Yong Chen, Gary W. Marek, Thomas H. Marek, Kevin R. Heflin, Dana O. Porter, Jerry E. Moorhead, David K. Brauer

**Affiliations:** 1 Department of Ecosystem Science and Management, Texas A&M University, College Station, TX 77843, USA; 2 USDA-ARS Conservation and Production Research Laboratory, 300 Simmons Rd., Unit 10, Bushland, TX 79012, USA; 3 Texas A&M AgriLife Research and Extension Center at Amarillo, 6500 Amarillo Blvd. W., Amarillo, TX 79106, USA; 4 Texas A&M AgriLife Research and Extension Center at Lubbock, 1102 E FM 1294, Lubbock, TX 79403, USA

**Keywords:** soil water sensor, soil water measurement, soil water content, neutron moisture meter, corn, semi-arid region, factory calibration, field correction

## Abstract

Performance evaluations and corrections of soil water sensors have not been studied using different installation orientations under various irrigation treatments in the Texas High Plains. This study evaluated the performance of four sensors using factory calibration and derived field corrections as compared to calibrated neutron moisture meters (NMMs). Sensor performance was assessed using horizontal insertion, laid horizontal placement, and vertical insertion at 15.2, 45.7, and 76.2 cm depths in a clay loam soil with three irrigation treatments. Results indicated the factory-calibrated Acclima 315 L performed satisfactorily using horizontal insertion as compared to NMM measurements at 45.7 and 76.2 cm depths with a ±2% mean difference (MD) and <3.5% root mean square error (RMSE). The factory-calibrated Acclima 315 L using horizontal insertion also performed satisfactorily across all irrigation treatments according to soil profile water storage (MD = 0.36% and RMSE = 3.25%). Generally, the factory-calibrated Decagon GS1 and Campbell Scientific 655 using vertical insertion agreed more closely with NMM measurements compared with other installation orientations. There was a significant underestimation of water storage (>60 mm) in the 0.9 m soil profile using the Watermark 200SS. In summary, field corrections are required for Decagon GS1, Campbell Scientific 655, and Watermark 200SS sensors.

## 1. Introduction

The semi-arid Southern Great Plains is one of the most productive irrigated agricultural regions in the United States (U.S.). The underlying Ogallala Aquifer serves as a crucial groundwater source for irrigated agricultural crop production. However, intensive irrigation pumping with very little recharge in this region has resulted in a considerable decline in groundwater storage of the Ogallala Aquifer in recent years [1,2]. In addition, competition for water from urban, industrial, and agricultural sectors along with a changing climate has resulted in notable droughts and water shortages, placing agricultural production and accompanying economic prosperity at risk [3]. Therefore, there is an urgent need for research regarding improved agricultural irrigation management practices for the U.S. Southern Great Plains.

Monitoring of soil water content (SWC) throughout the growing season can facilitate irrigation scheduling [4]. The use of soil water sensors for SWC estimations is gaining widespread state and federal support with cost sharing in the U.S. For instance, the U.S. Department of Agriculture (USDA) awarded the White River Irrigation District in Arkansas $4.45 million in 2009 to install soil water measurement and monitoring technologies, which included investments in soil water sensors [5]. In addition, several programs and networks were established in the U.S. for soil and climatic observations using different in situ sensors with differing accuracy and precision. For example, the Texas Soil Observation Network (TxSON), West Texas Mesonet, Oklahoma Mesonet, U.S. Climate Reference Network (USCRN), and Soil Climate Analysis Network (SCAN) are collecting SWC data using soil water sensors [6,7]. However, several studies have alluded that soil water sensors using factory calibrations report readings that do not adequately represent actual SWC in field conditions [8,9,10,11,12,13]. In most cases, a field or site-specific correction is required to improve the accuracy of sensor readings for agricultural and research applications [6]. Additionally, due to the vertical variations (stratification) of soil properties and water regimes, it is possible that the soil water sensors installed using different installation orientations may result in different SWC readings. For instance, Plauborg et al. [14] studied the effects of installation orientations of horizontal and vertical insertions on the performance of Campbell Scientific 616 (CS-616) and found that vertically installed CS-616 sensors responded well to changes in SWC when using the factory calibration. However, horizontally installed CS-616 sensors required a site-specific soil correction to obtain a reasonable measurement of SWC. Consequently, a soil-specific sensor correction may be necessary, depending on site-specific conditions and sensor application. However, a lack of such studies was found in the literature [15].

Corn (*Zea mays* L.) is one of the dominant crops in the semi-arid U.S. Southern Great Plains, which demands a large amount of irrigation water to meet its full crop evapotranspiration (ETc) requirement. Due to the declining groundwater resources and resultant reduced well capacities, deficit irrigation for corn production is increasingly being practiced [16,17,18,19,20], and accurate measurements of SWC are crucial for profitable corn production under limited irrigation strategies. As far as we know, concomitant comparisons of soil water sensor performance under different irrigation level treatments has rarely been studied [21]. In this study, the performance of a soil water potential sensor and three soil water content sensors installed at three depths and using three installation orientations under three irrigation treatments were evaluated against collocated measurements from a calibrated neutron moisture meter (NMM). The primary goal was to assess the accuracy of the soil water sensors using factory calibrations and determine field-based corrections, if needed. Specific objectives of the study were (1) to evaluate the accuracy of each of the four sensor types, installed using three different orientations at three depths, under three irrigation treatment levels, compared against SWC measurements from calibrated NMMs; (2) to compare the water storage in the soil profile derived from integrating the soil water sensor measurements against those integrated from NMM readings; and (3) to explore the field corrections for the four sensors and develop the soil-specific correction curves for use with regional agricultural production applications.

## 2. Materials and Methods

### 2.1. Study Area Description

**Figure 1:**
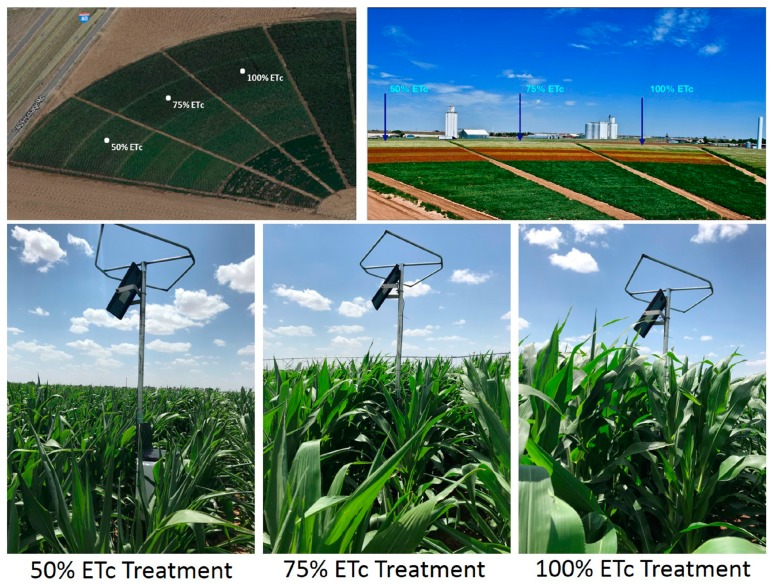
The Texas A&M AgriLife Research Emeny Center Pivot Field with corn under the three irrigation treatments of 100%, 75%, and 50% crop evapotranspiration (ETc) conditions.

The study site is located at the Texas A&M AgriLife Research Emeny Center Pivot Field, Bushland, Texas (35.2° N, 102.1° W). The elevation of the study site is ~1170 m above mean sea level, and the slope is <1%. The regional semi-arid climate has a mean annual precipitation and air temperature of 488 mm and 14.1 °C. The circular field is irrigated with a center pivot system equipped with low elevation spray application (LESA) nozzles positioned on a 1.5 m spacing and at a height of 0.5 m above the ground surface. Three targeted irrigation treatment levels of 100%, 75%, and 50% ETc requirements were used in this study (Figure 1). The soil is a Pullman clay loam soil (fine, mixed, superactive, thermic Torrertic Paleustoll) [22]. The permanent wilting point (PWP) and field capacity (FC) are approximately 0.18 and 0.33 m^3^ m^−3^, respectively. Measured site-specific soil information by layer is provided in Table S1 in the Supplementary Materials [23,24]. An adjacent research-grade weather station maintained in accordance with the American Society of Civil Engineers-Environmental and Water Resources Institute (ASCE-EWRI) specifications [25] was used to measure meteorological data for this study.

### 2.2. Agronomic Management

**Figure 2:**
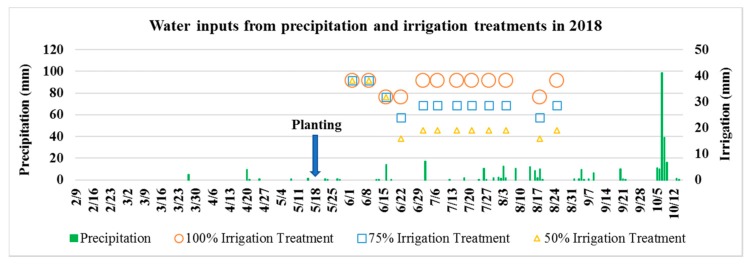
Precipitation and three irrigation treatments of 100%, 75%, and 50% crop evapotranspiration requirements in 2018.

Conventional tillage was performed prior to planting using a tandem disk. Fertilizer was applied according to recommendations using soil test data from a commercial soil testing company for a targeted corn grain yield of ~18.83 Mg ha^−1^ (300 bu ac^−1^). The field was bedded to a row spacing of 0.76 m (30 in.). A full-season corn hybrid, DuPont Pioneer 1366 AM (113 days—comparative relative maturity), was planted on 18 May 2018 at a rate of 83,275 seeds ha^−1^ (33,700 seeds ac^−1^) using a six-row John Deere computer controlled Max-Emerge planter (Deere & Co., Moline, IL, USA). Irrigations of 31.75 mm (1.25 in.) to 38.1 mm (1.5 in.) for each irrigation event were scheduled to target 100% of corn ET requirement using a growth-staged crop coefficient and daily reference ET method calculated from the meteorological station data. Deficit irrigation treatments of 75% and 50% ETc requirements occurred on the same dates but received 75% and 50% of the full irrigation amounts, achieved by increased system speed control (Figure 2). However, full irrigation of all three irrigation treatments was maintained during the early crop development period (30 days after planting) to ensure crop establishment and to satisfy water requirements of early vegetative growth stages. The corn was harvested on 15 November 2018.

### 2.3. Soil Water Sensor Installation and Layout

Three trenches measuring 0.5 m (20 in.) wide by 5.5 m (18 ft.) long by 0.76 m (30 in.) deep were excavated in each of the irrigation treatment fields in January 2018. Each trench was centered between two crop beds to allow for installation of the soil sensors on each side of the trench. Sensors were installed at depths of 15.2 cm (6 in.), 45.7 cm (18 in.), and 76.2 cm (30 in.), the midpoints of soil profile depth zones of 0–0.30 m (0–1 ft.), 0.30–0.61 m (1–2 ft.), and 0.61–0.91 m (2–3 ft.). Subsequent surface tillage and bedding were conducted by hand to avoid damage to the 15.2 cm sensors and wiring. Care was taken so that manual tillage and shaping operations would match those of the surrounding field. At planting, corn seed was manually planted at double the field seeding rate in the sensor array area to ensure adequate germination. Following germination, plants in the sensor array area were thinned manually to match the populations of the surrounding fields so that water extraction would be similar. Four commercially available sensors were chosen for evaluation. A soil water potential sensor (Watermark 200SS, Irrometer, Inc., Riverside, CA, USA) and three soil water content sensors, Acclima 315L (Acclima, Inc., Meridian, ID, USA), Decagon GS1 (METER Group, Inc., Pullman, WA, USA), and Campbell Scientific 655 (Campbell Scientific, Inc., Logan, UT, USA), denoted as WM-200SS, ACC-315L, DEC-GS1, and CS-655, respectively, hereafter, were installed and became operational on 8 February 2018.

Sensor installation orientations consisted of vertical insertion into undisturbed soil, laid horizontal placement, and horizontal insertion into undisturbed soil (Figure S1 in the Supplementary Materials). The laid horizontal placement was performed by placing the sensor at the prescribed depths over undisturbed soil, either at the bottom of the trench (76.2 cm) or over shelves cut into the trench sidewalls (15.2 and 45.7 cm), followed by manually backfilling soil over the sensor. The horizontal insertion orientation for sensors with rod electrodes was achieved by inserting the sensor into the undisturbed soil sidewall at each of the three depths. Vertical insertion was achieved by inserting sensors into undisturbed soil into shelves cut into the trench sidewalls. Vertical and horizontal insertion of the WM-200SS sensors was achieved by boring a slightly oversized hole into undisturbed soil, followed by insertion using a soil slurry. It is acknowledged that the manufacturers of the WM-200SS do not recommend installation orientations other than vertical. However, all installation orientations were performed for all sensor types to address questions from area producers and crop consultants regarding sensor installation procedures and orientations and potential effects on output values. Duplicate sensors for each sensor type, orientation, and depth were installed in each trench at each location. The average of the two sensor values from each replication was used to conduct the analyses in this study. In the case of erroneous or missing data, a singular value from one sensor was used for analysis. Seventy-two sensors in total were installed (18 each of the four study sensors) in each trench, resulting in sensors being positioned approximately every 20 cm horizontally along the trench sidewalls. The trench was backfilled and water packed using the excavated soil. Crop beds and furrows were then manually constructed within the trench area to align them with those of the circular rows of the field. Sensors were polled every five minutes and data were compiled into 15-min average values. The data collection instrumentation consisted of a Campbell Scientific CR6 datalogger coupled with an AM 16/32 multiplexer for each of the three treatment plots.

Two NMM access tubes were installed adjacent to each end of the sensor array in undisturbed soil for each ETc level. Readings from the NMM were used for comparison with values from the soil water sensors in this study. In general, the NMM readings were taken twice weekly, one or two days prior to an irrigation event, and two days following an irrigation or precipitation. This approach was designed to capture SWC readings from both relatively wet and dry soil conditions.

### 2.4. Sensor Performance Comparisons

Sensor performance was assessed by comparing statistical parameters using two approaches: (1) Comparison between the average SWC of the sensor pairs and the NMM measurements at each installation depth (i.e., point comparisons), and (2) comparison of water storage in the soil profile as determined by integration of soil water sensor values and the NMM measurements. Direct comparison between depth-specific sensor measurements and NMM readings is known to be problematic. Bell et al. [26] and Schwartz et al. [27] cautioned against making direct comparisons between depth-specific SWC readings from NMMs and collocated soil water sensor measurements due to the large differences in the sensing volume of the NMM (2.8–3.4 × 10^5^ cm^3^) compared to that of most soil water sensors (<500 cm^3^). This difference in soil sensing volumes can result in significantly different SWC readings as relatively small inconsistencies in soil properties and conditions can be overstated by a small sensing volume, but lessened, or averaged out by a larger sensing volume. Therefore, point measurements from the soil sensor array were integrated to estimate the profile water storage for comparison with the corresponding NMM measurements.

In this study, water storage of the soil profile to a depth of 0.9 m was determined by integrating both soil water measurements of the respective sensor at the three depths and from NMM readings at depths of 0.1, 0.3, 0.5, and 0.7 m with using a half weighted value for the 0.9 m depth [27,28]. Specifically, the soil profile water storage determined by NMM readings was calculated using 2/9 weighted value at the depths of 0.1, 0.3, 0.5, and 0.7 m and using a 1/9 weighted value of the 0.9 m depth. A depth of 0.9 m is generally deemed as the manageable irrigation profile depth during the growing season for the clay loam soil prevalent within the region. Statistical analysis included descriptive statistics and analyses of variance (*p* < 0.05) conducted using SPSS 22.0 software (IBM SPSS Statistics, IBM Corporation, Armonk, NY, USA) to detect differences between sensor and NMM measurements. Three statistical parameters were used to evaluate the performance of the soil water sensors as compared to the NMM measurements.

The mean difference (MD) is the average difference between sensor and NMM measurements [29], calculated as:

(1) $$\mathrm{MD}=\frac{\sum_{i=1}^{n}\left(\mathrm{Msi}-\mathrm{Mni}\right)}{n}$$

where M_si_ is the ith measurement obtained through a sensor; M_ni_ is the ith measurement acquired by a NMM; and n is the sample size.

The root mean square error (RMSE) is the total difference between a sensor and the corresponding NMM measurements of the soil water content [30], calculated as:

(2) $$\mathrm{RMSE}=[\frac{1}{n}\sum_{i=1}^{n}{\left(\mathrm{Msi}-\mathrm{Mni}\right)}^{2}{]}^{0.5}$$

The standard deviation (SD) is a measure that is used to quantify the amount of variation or dispersion of a set of data values [31], calculated as:

(3) $$\mathrm{SD}=\sqrt[{2}]{{\mathrm{RMSE}}^{2}-{\mathrm{MD}}^{2}}$$

The coefficient of determination (*R*^2^) was used to demonstrate the degree of association between sensor measurements and NMM readings. Linear regression was performed to compute the slope and intercept values [32,33]. Linear, exponential, logarithmic, and quadratic correction equations were used for the sensor field corrections. Values of MD, RMSE, and SD approaching zero and *R*^2^ approaching a value of 1 indicate good agreement between the sensor values and NMM measurements.

## 3. Results and Discussion

### 3.1. Vertical Soil Water Dynamics and Water Storage in the Soil Profile Using Neutron Moisture Meters

**Figure 3:**
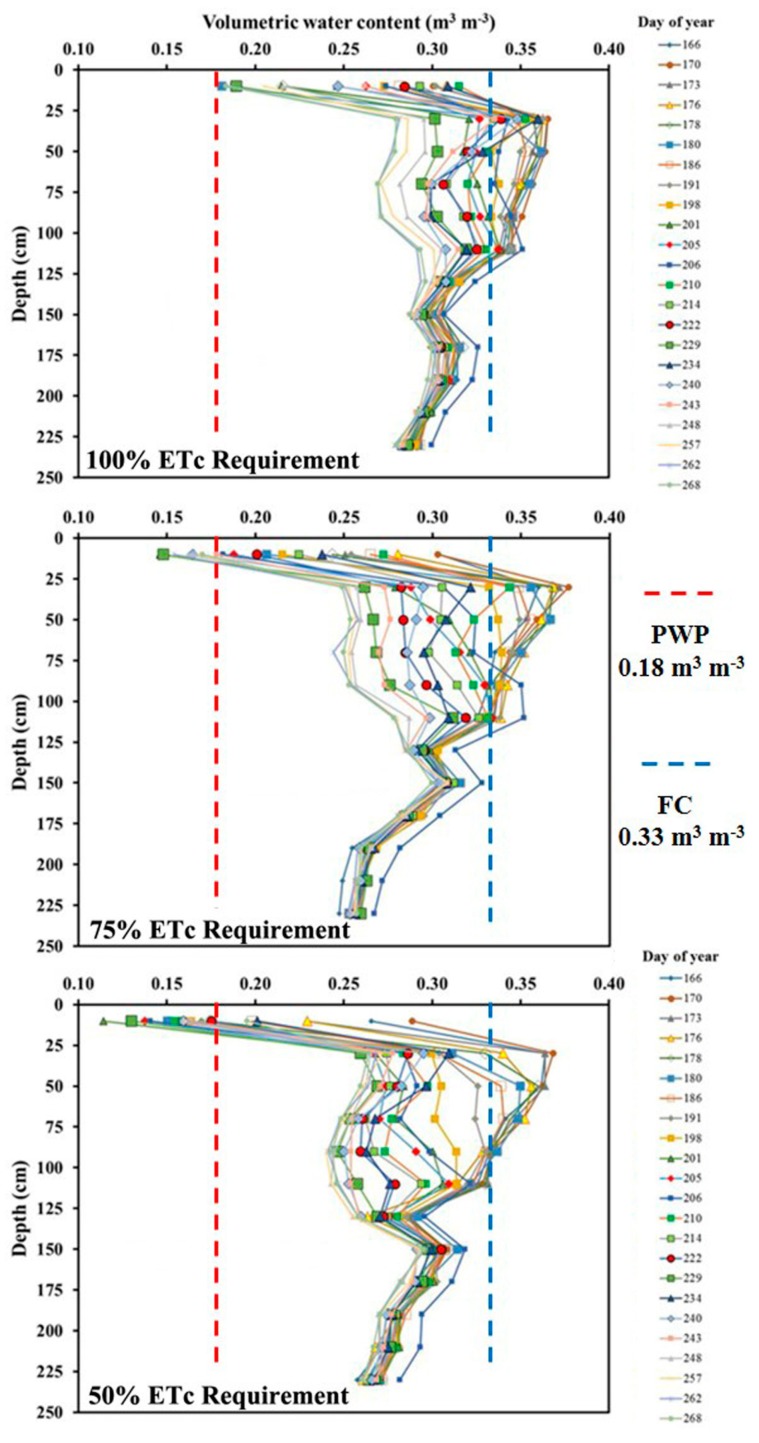
Volumetric soil water content (m^3^ m^−3^) at the depths of 10, 30, 50, 70, 90, 110, 130, 150, 170, 190, 210, and 230 cm using the neutron moisture meters for days during the corn growing season under 100%, 75%, and 50% crop evapotranspiration (ETc) requirements.

Volumetric SWC measured by the NMMs ranged from 0.11 to 0.31 m^3^ m^−3^ at the 10-cm depth during the corn growing season under three irrigation treatments (Figure 3). Conventional tillage in this study increased soil evaporation potential and deepened the evaporative drying front to at least 10 cm, which resulted in large variation in SWC near the soil surface under irrigation conditions [34]. This is expected for a study being conducted in a high ET demand environment. The average annual standardized alfalfa reference ET (ETrs) is ~1600 mm within the region [25]. The three irrigation treatments captured the wet and dry soil conditions given the PWP and FC of 0.18 and 0.33 m^3^ m^−3^, respectively (Figure 3). It was expected that the 100% ETc treatment demonstrated relatively high SWC compared to the 75% and 50% ETc treatments. From depths 30 to 130 cm, the SWC varied from 0.24 to 0.38 m^3^ m^−3^. Smaller variations of 0.24 to 0.33 m^3^ m^−3^ in SWC were observed below the 150-cm depth for the Pullman clay loam soil (Figure 3). Integrated water storage in the 0.9 m soil profiles varied from 229 to 312 mm, 207 to 310 mm, and 207 to 308 mm with average values of 280, 261, and 243 mm, respectively, under the 100%, 75%, and 50% irrigation treatments (Table 1).

### 3.2. Soil Water Sensor Performance Using Factory Calibration

Factory calibrated daily SWC values for each sensor type, depth, and installation orientation were calculated and compared with NMM measurements from similar depths during the corn growing season. In general, soil water sensor measurements, irrespective of installation orientation and irrigation treatment, were significantly different from the NMM measurements (*p* < 0.05) with relatively high SD values >2.45% at the 15.2 cm depth, with the exception of the WM-200SS sensor (Table 2). Varble and Chávez [21] also found that the factory calibrated Acclima TDT, Decagon 5TE, and Campbell Scientific 616/625 sensors performed unsatisfactorily at a depth of 10 cm when compared to periodic thermogravimetrically determined SWC in a sandy clay loam soil in Colorado. According to Hignett and Evett [8], statistical goals of MD within ±2% and RMSE < 3.5% for sensor applications in agricultural production and research are acceptable. Recently, Datta et al. [35] reported that the ACC-315L, DEC-GS1, and CS-655 sensors with factory calibration performed unsatisfactorily at the 20 cm depth when compared to thermogravimetric SWC measurements in a soil with a high level of clay content in Oklahoma. Differences between the NMM and soil water sensor measurements at the 15.2 cm depth were also observed, likely because the NMM does not typically perform well for measurement depths near the surface layer [26,27]. In addition, voids at the near surface layer can affect the sensor measurements.

In general, the ACC-315L sensor performed satisfactorily using the horizontal insertion with the factory calibration as compared to NMM measurements at the 45.7 and 76.2 cm depths for all three irrigation treatments, according to the Hignett and Evett [8] statistical goals (Table 3 and Table 4). The ACC-315L sensors also captured most of the soil water variation using the horizontal insertion orientation (*R*^2^ > 0.73) at the 45.7 and 76.2 cm depths, with relatively small SD values <3.21%. The ACC-315L displayed the regression slopes that approximated the 1:1 line, with a range of 0.97 to 1.61 under the three irrigation treatments. In general, the factory-calibrated DEC-GS1 sensor using vertical insertion performed better than other installation orientations for the depths of 45.7 and 76.2 cm according to the MD and RMSE values. However, no consistent trend regarding installation orientation was determined for the three irrigation treatments in the comparisons of the DEC-GS1 sensor with the corresponding NMM measurements. As such, field corrections are warranted for use in agricultural applications. Generally, the CS-655 sensor using vertical insertion outperformed other installation orientations at the 45.7 and 76.2 cm depths for 100% (wet) and 50% (dry) irrigation treatments (Table 3 and Table 4). However, further corrections were needed when using the CS-655 sensor for agricultural and research applications [8]. Significant MD (*p* < 0.05) and large RMSE were observed for the WM-200SS sensors at the depths of 45.7 and 76.2 cm with the three irrigation treatments using the factory calibration irrespective of installation orientation (Table 3 and Table 4).

### 3.3. Comparison of Integrated Soil Profile Water Storage Using Factory Calibration under Three Irrigation Treatments

**Figure 4:**
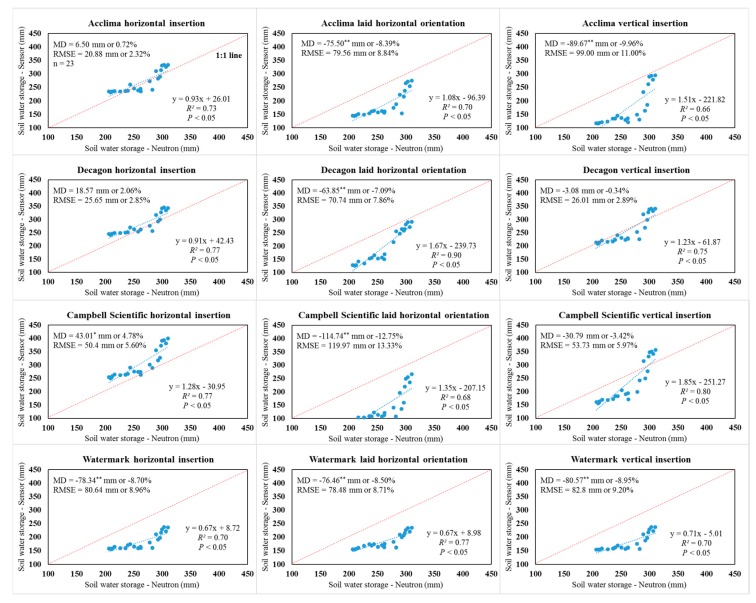
Graphical and statistical comparisons of sensor and neutron moisture meter-derived soil profile water storage values in the upper 0.9 m soil profile under the 75% crop evapotranspiration treatment. MD indicates mean difference; RMSE indicates root mean square error; * indicates a significant difference at *p* < 0.05; ** indicates a significant difference at *p* < 0.01.

**Figure 5:**
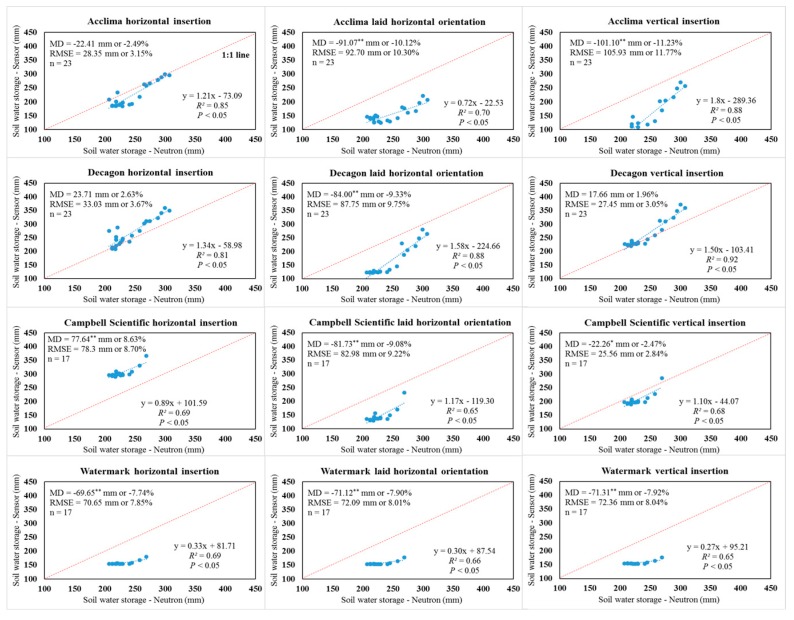
Graphical and statistical comparisons of sensor and neutron moisture meter-derived soil profile water storage values in the upper 0.9 m soil profile under the 50% crop evapotranspiration treatment. MD indicates mean difference; RMSE indicates root mean square error; * indicates a significant difference at *p* < 0.05; ** indicates a significant difference at *p* < 0.01.

Comparison of water storage in the 0.9 m soil profile integrated from both the soil water sensors and NMM measurements indicated the factory-calibrated ACC-315L sensors performed satisfactorily using the horizontal insertion orientation under the 75% ETc treatment (RMSE = 20.88 mm and 2.32% and MD = 6.50 mm or 0.72%), according to the Hignett and Evett [8] statistical goals (Figure 4). In addition, the DEC-GS1 sensor using vertical insertion did not require correction under the 75% and 50% ETc treatments. The MD, RMSE, *R*^2^, and slope were −0.34% and 1.96%, 2.89% and 3.05%, 0.75 and 0.92, and 1.23 and 1.50 for the vertically inserted DEC-GS1 sensors under the 75% and 50% ETc treatments, respectively (Figure 4 and Figure 5). The 75% irrigation treatment represents the dominant “deficit irrigation strategy” used with limited well capacities in the Texas High Plains. This management level is necessitated by the reduced well capacity as well as regional groundwater district regulation (pumping limits). The satisfactory performance of the factory-calibrated ACC-315L and DEC-GS1 sensors for specific installation orientations for the 75% ETc treatment may provide useful information for certain producers, consultants, and extension specialists. Similar to the CS-655 sensor comparison at depths, the associated soil profile water storage values integrated from CS-655 sensors using vertical insertion outperformed other installation orientations but needed correction for agricultural applications under all three irrigation treatments (Figure S2, Figure 4 and Figure 5). Again, significant MD and large RMSE were found with the WM-200SS sensors. The WM-200SS sensors significantly underestimated water storage within the 0.9 m soil profile when compared to the NMM values under the three irrigation treatments (Figure S2, Figure 4 and Figure 5).

**Figure 6:**
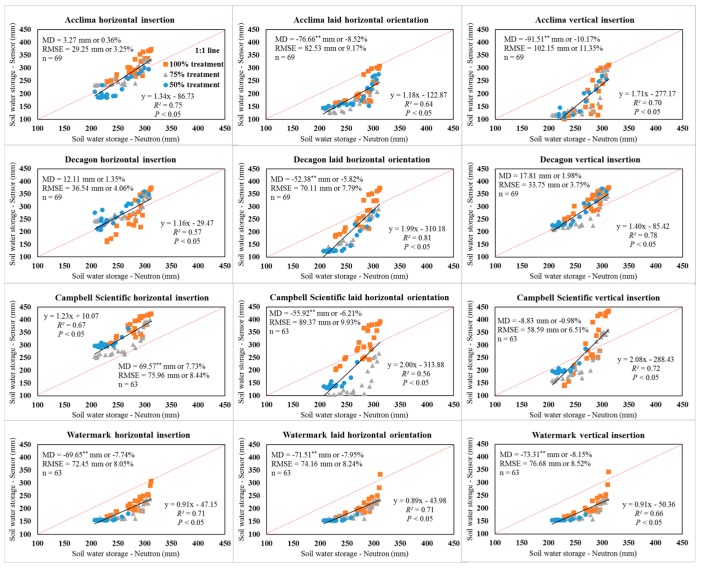
Graphical and statistical comparisons of sensor and neutron moisture meter-derived soil profile water storage values in the upper 0.9 m soil profile when combining all the data from three irrigation treatments. MD indicates mean difference; RMSE indicates root mean square error; ** indicates a significant difference at *p* < 0.01.

As shown in Figure 6, pooled data (n = 69) from all irrigation treatments for the ACC-315L sensors using horizontal insertion were acceptable for use in agricultural production and research settings using the factory calibration. In view of the MD of soil water storage in the profile, the DEC-GS1 sensors using the horizontal and vertical insertions might also be used without correction (Figure 6). However, the MD of the sensor comparison of individual depths indicated corrections were required for the DEC-GS1 sensor for agricultural applications in all three irrigation treatments. Therefore, the Hignett and Evett [8] statistical goals of “MD within ±2% and RMSE < 3.5%” were more suitable for a specific depth or point comparison. As for comparisons for profile water storage values, the statistical goals of “MD within ±1% and RMSE < 3.5%” were proposed using soil water sensors in irrigation scheduling and related agricultural applications. Therefore, DEC-GS1 sensors required corrections based on the proposed statistical goals. Results showed readings from the ACC-315L sensor were also sensitive to installation orientation. Soil water storage was significantly underestimated by ACC-315L sensors using both the laid horizontal and vertical insertion orientations under all three irrigation treatments (Figure 6). Measurements from the TDR-based ACC-315L should not be affected by sensor orientation. However, differences in soil bulk density attributed to the manually packed (laid horizontal) and undisturbed (horizontal and vertical) insertion likely influenced measurements. In general, the soil was less distributed under the horizontal insertion as compared to the laid horizontal orientation, resulting in higher bulk densities and reasonable agreement with NMM readings (Figure 4). The differences associated with horizontal and vertical insertion orientations are more difficult to explain as they were also installed in undisturbed soil. It is uncertain if measurements were affected by a vertical soil temperature gradient, as temperature was not integrated with depth, but was measured at a single point by a singular thermistor located just inside the sensor head in the outer waveguide. The ACC-315L integrated water content along the length of the probe, resulting in a mean water content. These may partially explain the difference in values associated with the installation orientations, particularly for the sensors positioned at the 15.2 cm depth as a considerable SWC gradient may exist near the soil surface following soil wetting events. The CS-655 sensor tended to overestimate the profile water storage under relatively wet conditions (280–330 mm) with deviations from the 1:1 line being large, with slopes higher than two using the laid horizontal and vertical insertion orientations. Even though significant MD was identified for the WM-200SS sensors, the slopes were all close to one, which indicated a systemic underestimation of the soil water storage when using the WM-200SS sensors, regardless of the installation orientation and irrigation treatment (Figure 6).

### 3.4. Field Corrections of Integrated Soil Profile Water Storage

Results showed that all soil water sensors could be used for irrigation scheduling in this study region when used with a field correction based on the proposed statistical goals. As for the field correction equations for soil profile water storage, the quadratic equation had the highest *R*^2^ for the four sensors (Table 5). It is worth noting that all field correction equations used in this study for the DEC-GS1 sensors using the horizontal insertion resulted in an *R*^2^ < 0.65, which generally would not be deemed acceptable. Therefore, in addition to the MD and RMSE, we recommend the adopted fitting equation to have an *R*^2^ > 0.65 for irrigation scheduling purposes and associated applications. The suggested evaluation criteria for soil water sensor performance, therefore, is MD within ±1%, RMSE < 3.5%, and *R*^2^ > 0.65 for data fitting using soil profile water storage (Table 5). According to these evaluation criteria, the DEC-GS1 sensors using a vertical insertion may be appropriate for agricultural water management within this study region when using the field correction equations developed from this study data. It is also worth noting that the field-corrected CS-655 sensors in this study had larger *R*^2^ values using the vertical insertion relative to those using horizontal insertion (Table 5). Assuming a vertical orientation represents a more integrated value in terms of the soil profile layers useful for irrigation management.

As for the relationship between correction equations and installation orientations, the DEC-GS1 sensors using the horizontal and vertical insertions had the closest equation coefficients (“a” and “b”) when using the linear and exponential equation types (Table 5). However, small *R*^2^ values were evident with the horizontal insertion orientation. The relatively short electrodes of these sensors may affect the “integrating potential” as compared to sensors with longer waveguides. The apparent differences of the equation coefficients are shown for all equation types when comparing CS-655 sensors using horizontal insertion against vertical insertion. Regarding the WM-200SS sensors, the three installation orientations exhibited close equation coefficients for all equation types. Overall, the ACC-315 L, DEC-GS1, and CS-655 sensors were sensitive to installation orientation, and different field correction equations may need to be used with the respective installation orientation. As shown in Table 5, the WM-200SS sensor was not susceptible to installation orientation, which provides flexibility in sensor installation. However, field-specific correction is required for agricultural applications. We acknowledge there are some limitations of this study. It is generally suggested that installation orientation should not affect sensor readings, but data in this study using these installation processes suggested otherwise. 

These are preliminary data from the first year of the study, and the disturbed soil (due to installation) may need time to consolidate. It may also require a “water packing effect (generally achieved through multiple irrigations or heavy rains)” to adequately settle the soil in the trench even though the sensors were installed in February 2018. While this settling period is recognized and necessary, installation according to the manufacturer’s recommendations would also require a bore hole or equivalent entry port to install the sensors in the horizontal orientation, particularly at deeper profile depths. In addition, the spatial and depth matching of the sensor and NMM measurements have a computational impact on the comparison values on the soil water in a management layer. Finally, direct calibration of the sensor-measured apparent permittivity may be necessary when more data are available from subsequent study years. This applied research addresses how actual producers could use the soil water sensors for irrigation scheduling, which may or may not follow manufacturer recommendations regarding the installation, interpretation, and use of sensor readings. The results in this study provide information of the performance of various soil water sensors under different irrigation treatments with multiple installation orientations and offer soil-specific correction equations for the heavier soils of the Texas High Plains.

## 4. Conclusions

This study evaluated the performance of ACC-315L, DEC-GS1, CS-655, and WM-200SS soil sensors in multiple field-based water regimes between PWP and FC using various installation orientations with the factory calibration and field correction in a Pullman clay loam soil using NMM readings as a control reference. Overall, good agreement between sensor measurements and NMM readings was observed at the depths of 45.7 and 76.2 cm under the three irrigation treatments. Sensor installation orientation had a large impact on the performance of most sensors. In general, the horizontal and vertical insertions with less soil disturbance yielded sensor readings that were more representative of field water contents compared with the laid horizontal orientation. The factory-calibrated ACC-315L performed satisfactorily using the horizontal insertion installation at depths of 45.7 and 76.2 cm with values of RMSE < 3.5%, SD < 2.4%, and MD within ±2%. Also, it performed optimally in terms of soil profile water storage using a horizontal insertion orientation across all irrigation treatments (RMSE = 29.25 mm or 3.25% and MD = 3.27 mm or 0.36%). Satisfactory performance of the factory-calibrated ACC-315L sensors with a horizontal insertion orientation can be used and benefit local/regional producers, who may not be willing to use a field correction. This research also suggests that field correction is required for the DEC-GS1, CS-655, and WM-200SS sensors to be used in irrigation scheduling. One linear and three non-linear field correction equations were reported for possible use by crop/irrigation consultants, extension professionals, and relatively experienced soil water sensor users and producers to correct the soil water sensors for use in regions with a similar soil. Finally, according to the sensor comparison of depths and comparison of soil profile water storage, we suggest the sensor performance statistical goals of MD within ±2%, RMSE < 3.5%, and *R*^2^ > 0.65 for data fitting can be used in specific depth comparisons. As for the comparison of soil profile water storage, the evaluation criteria for sensor performance of MD within ±1%, RMSE < 3.5%, and *R*^2^ > 0.65 for data fitting is recommended in most agricultural and research applications. This sensor performance assessment and correction study also serves to potentially provide statistically-based soil water sensor recommendations for the USDA-NRCS agency guidelines as well as that of other water management and water policy agencies.

## Figures and Tables

**Table 1:** Water storage in the 0.9 m soil profiles (mm) using the neutron moisture meters for three irrigation treatments.

Day of Year (DOY)	Date	100% ETc Treatment	75% ETc Treatment	50% ETc Treatment
166	6/15/2018	307.1	303.5	299.7
170	6/19/2018	312.4	310.2	307.7
173	6/22/2018	297.3	299.7	294.0
176	6/25/2018	310.7	306.7	288.4
178	6/27/2018	291.5	298.0	275.1
180	6/29/2018	282.3	289.3	265.7
186	7/5/2018	300.8	296.4	269.3
191	7/10/2018	306.1	292.6	257.7
198	7/17/2018	293.4	278.4	245.3
201	7/20/2018	268.9	244.2	219.0
205	7/24/2018	279.5	250.9	218.8
206	7/25/2018	292.4	262.1	229.6
210	7/29/2018	296.0	282.8	229.8
214	8/2/2018	283.3	257.8	221.0
222	8/10/2018	281.8	239.8	226.3
229	8/17/2018	247.9	216.5	207.2
234	8/22/2018	289.2	262.6	241.3
240	8/28/2018	273.0	235.9	224.1
243	8/31/2018	271.2	226.7	218.7
248	9/5/2018	246.4	212.3	212.7
257	9/14/2018	237.4	211.3	212.9
262	9/19/2018	229.9	206.8	212.5
268	9/25/2018	229.4	209.8	217.8
Minimum value		229.4	206.8	207.2
Maximum value		312.4	310.2	307.7
Average value		279.5	260.6	243.2

**Table 2:** Statistical summary of sensor performance at the 15.2 cm depth.

Sensor (Treatment)	Installation Orientation	MD (%)	RMSE (%)	SD (%)	Slope	Intercept	*R* ^2^
	**Acclima 315L (n = 23)**						
100% ETc	Horizontal insertion	7.51 ^a^	9.43	5.69	0.68 ^b^	15.62 ^c^	0.23
	Laid horizontal	−7.37 ^a^	8.66	4.54	0.43 ^b^	7.06	0.20
	Vertical insertion	−6.45 ^a^	9.26	6.65	1.26 ^b^	−12.99	0.42
75% ETc	Horizontal insertion	5.84 ^a^	6.84	3.56	0.62 ^b^	14.04 ^c^	0.50
	Laid horizontal	−1.66	3.47	3.05	0.62 ^b^	6.53 ^c^	0.62
	Vertical insertion	−4.47 ^a^	5.82	3.72	0.73 ^b^	1.23	0.52
50% ETc	Horizontal insertion	−2.32 ^a^	4.79	4.19	0.32	9.70 ^c^	0.16
	Laid horizontal	−3.50 ^a^	4.26	2.45	0.51 ^b^	5.21 ^c^	0.70
	Vertical insertion	−4.97 ^a^	5.87	3.11	0.74 ^b^	−0.27	0.52
	**Decagon GS1 (n = 23)**						
100% ETc	Horizontal insertion	6.29 ^a^	8.25	5.33	1.49 ^b^	−6.21	0.65
	Laid horizontal	6.66 ^a^	7.65	3.76	1.07 ^b^	4.96	0.61
	Vertical insertion	7.31 ^a^	8.22	3.78	0.73 ^b^	14.13 ^c^	0.45
75% ETc	Horizontal insertion	3.63 ^a^	4.85	3.21	0.60 ^b^	12.17 ^c^	0.58
	Laid horizontal	2.57	3.80	2.79	1.03 ^b^	1.89	0.77
	Vertical insertion	12.68 ^a^	13.04	3.04	0.54 ^b^	22.45 ^c^	0.63
50% ETc	Horizontal insertion	7.51 ^a^	8.00	2.74	0.75 ^b^	11.92 ^c^	0.60
	Laid horizontal	−2.47 ^a^	4.93	4.26	1.05 ^b^	−3.29	0.51
	Vertical insertion	7.35 ^a^	8.14	3.50	1.04 ^b^	6.72	0.60
	**Campbell Scientific 655**						
100% ETc (n = 23)	Horizontal insertion	13.71 ^a^	14.43	4.49	0.56 ^b^	24.84 ^c^	0.27
	Laid horizontal	6.57 ^a^	8.53	5.44	0.52 ^b^	18.85 ^c^	0.17
	Vertical insertion	8.14 ^a^	11.62	8.30	1.39 ^b^	−1.70	0.36
75% ETc (n = 23)	Horizontal insertion	7.26 ^a^	8.53	4.48	1.10 ^b^	5.07 ^c^	0.60
	Laid horizontal	−4.65 ^a^	5.54	3.01	0.74 ^b^	0.96	0.64
	Vertical insertion	14.82 ^a^	15.26	3.64	0.87 ^b^	17.59 ^c^	0.59
50% ETc (n = 17)	Horizontal insertion	11.85 ^a^	12.28	3.25	0.37	22.08 ^c^	0.074
	Laid horizontal	1.43	3.05	2.69	0.37	11.54 ^c^	0.12
	Vertical insertion	−2.20 ^a^	4.62	4.06	0.59	4.48	0.10
	**Watermark 200SS**						
100% ETc (n = 23)	Horizontal insertion	−1.58	4.19	3.88	0.50 ^b^	11.20 ^c^	0.33
	Laid horizontal	−2.28	3.78	3.01	0.54 ^b^	9.29 ^c^	0.54
	Vertical insertion	−1.22	4.31	4.13	0.48 ^b^	11.88 ^c^	0.28
75% ETc (n = 23)	Horizontal insertion	−1.97	3.97	3.44	0.41 ^b^	10.73 ^c^	0.55
	Laid horizontal	−0.18	3.40	3.40	0.42 ^b^	12.15 ^c^	0.56
	Vertical insertion	−1.52	3.65	3.32	0.44 ^b^	10.54 ^c^	0.58
50% ETc (n = 17)	Horizontal insertion	−1.32	2.48	2.09	0.087	16.07 ^c^	0.19
	Laid horizontal	1.37	2.49	2.08	0.12	15.57 ^c^	0.16
	Vertical insertion	1.08	2.41	2.15	0.05	16.42 ^c^	0.18

^a^ MD (mean difference) is significantly different from zero at *p* < 0.05 level. ^b^ Slope was significantly different from zero at *p* < 0.05 level. ^c^ Intercept is significantly different from zero at *p* < 0.05 level. RMSE indicates root mean square error. SD indicates standard deviation.

**Table 3:** Statistical summary of sensor performance at the 45.7 cm depth.

Sensor (Treatment)	Installation Orientation	MD (%)	RMSE (%)	SD (%)	Slope ^b^	Intercept	*R* ^2^
	**Acclima 315L (n = 23)**						
100% ETc	Horizontal insertion	2.04	3.17	2.42	1.36	−9.92	0.73
	Laid horizontal	−3.92 ^a^	6.40	5.07	2.19	−43.18 ^c^	0.70
	Vertical insertion	−9.35 ^a^	12.25	7.91	2.77	−67.68 ^c^	0.58
75% ETc	Horizontal insertion	−0.75	2.36	2.23	1.09	−3.61	0.79
	Laid horizontal	−8.44 ^a^	9.89	5.16	1.22	−15.44	0.47
	Vertical insertion	−12.51 ^a^	14.31	6.95	2.08	−46.27 ^c^	0.69
50% ETc	Horizontal insertion	0.088	2.04	2.04	1.28	−8.55 ^c^	0.89
	Laid horizontal	−9.76 ^a^	10.38	3.54	0.77	−2.80	0.42
	Vertical insertion	−16.17 ^a^	17.48	6.62	2.35	−57.34 ^c^	0.82
	**Decagon GS1 (n = 23)**						
100% ETc	Horizontal insertion	−0.50	5.79	5.77	2.73	−57.50 ^c^	0.83
	Laid horizontal	−2.74	6.87	6.30	2.52	−52.69 ^c^	0.67
	Vertical insertion	5.03 ^a^	5.57	2.38	1.31	−5.10	0.71
75% ETc	Horizontal insertion	3.19 ^a^	3.84	2.14	1.01	2.79	0.78
	Laid horizontal	−3.84 ^a^	6.08	4.71	1.80	−28.80 ^c^	0.80
	Vertical insertion	−3.77	4.82	3.02	1.35	−14.67 ^c^	0.80
50% ETc	Horizontal insertion	−0.21	5.06	5.05	1.67	−20.56 ^c^	0.68
	Laid horizontal	−8.31 ^a^	8.59	2.16	1.08	−10.65 ^c^	0.78
	Vertical insertion	−0.58	3.08	3.02	1.42	−13.27 ^c^	0.81
	**Campbell Scientific 655**						
100% ETc (n = 23)	Horizontal insertion	8.57 ^a^	9.03	2.86	1.65	−12.70	0.79
	Laid horizontal	0.62	5.03	4.99	2.34	−43.45 ^c^	0.77
	Vertical insertion	2.90	9.36	8.90	3.93	−93.39 ^c^	0.87
75% ETc (n = 23)	Horizontal insertion	6.54 ^a^	6.89	2.16	1.09	3.76	0.80
	Laid horizontal	−15.49 ^a^	15.77	2.95	0.96	−14.18 ^c^	0.62
	Vertical insertion	−5.76 ^a^	9.62	7.71	2.30	−46.69 ^c^	0.71
50% ETc (n = 17)	Horizontal insertion	9.61 ^a^	9.72	1.42	0.39	27.03 ^c^	0.77
	Laid horizontal	−13.08 ^a^	13.31	2.43	1.43	−25.40 ^c^	0.66
	Vertical insertion	−0.94	1.82	1.56	0.32	18.37 ^c^	0.69
	**Watermark 200SS**						
100% ETc (n = 23)	Horizontal insertion	−8.69 ^a^	9.05	2.56	1.29	−18.22 ^c^	0.67
	Laid horizontal	−9.16 ^a^	10.06	4.16	1.52	−26.39 ^c^	0.53
	Vertical insertion	−10.01 ^a^	10.49	3.11	1.52	−27.12 ^c^	0.68
75% ETc (n = 23)	Horizontal insertion	−11.01 ^a^	11.22	2.16	0.73	−2.57	0.70
	Laid horizontal	−11.85 ^a^	12.08	2.34	0.64	−0.48	0.65
	Vertical insertion	−11.67 ^a^	11.92	2.41	0.74	−3.35	0.64
50% ETc (n = 17)	Horizontal insertion	−11.10 ^a^	11.24	1.75	0.22	11.15 ^c^	0.65
	Laid horizontal	−11.26 ^a^	11.41	1.81	0.19	11.89 ^c^	0.60
	Vertical insertion	−11.13 ^a^	11.28	1.81	0.18	12.18 ^c^	0.67

^a^ MD (mean difference) is significantly different from zero at *p* < 0.05 level. ^b^ Slope was significantly different from zero at *p* < 0.05 level. ^c^ Intercept is significantly different from zero at *p* < 0.05 level. RMSE indicates root mean square error. SD indicates standard deviation.

**Table 4:** Statistical summary of sensor performance at the 76.2 cm depth.

Sensor (Treatment)	Installation orientation	MD (%)	RMSE (%)	SD (%)	Slope ^b^	Intercept	*R* ^2^
	**Acclima 315L (n = 23)**						
100% ETc	Horizontal insertion	2.02	2.60	1.64	1.47	−12.84 ^c^	0.96
	Laid horizontal	−6.85 ^a^	8.28	4.65	2.06	−40.68 ^c^	0.75
	Vertical insertion	−9.12 ^a^	11.14	6.39	2.52	−57.56 ^c^	0.72
75% ETc	Horizontal insertion	0.42	1.87	1.83	0.97	1.31	0.79
	Laid horizontal	−11.73 ^a^	12.14	3.13	1.15	−16.40 ^c^	0.65
	Vertical insertion	−9.57 ^a^	10.74	4.87	1.47	−23.90 ^c^	0.58
50% ETc	Horizontal insertion	−1.52	3.55	3.21	1.61	−19.29 ^c^	0.90
	Laid horizontal	−13.38 ^a^	13.60	2.47	0.53	0.36	0.63
	Vertical insertion	−8.83 ^a^	9.48	3.45	1.68	−28.57 ^c^	0.90
	**Decagon GS1 (n = 23)**						
100% ETc	Horizontal insertion	−4.78 ^a^	8.46	6.97	2.76	−61.14 ^c^	0.75
	Laid horizontal	−4.03	7.95	6.85	2.79	−61.10 ^c^	0.78
	Vertical insertion	3.60	4.39	2.51	1.40	−9.28	0.78
75% ETc	Horizontal insertion	2.71 ^a^	3.27	1.83	1.01	2.43	0.80
	Laid horizontal	−16.67 ^a^	17.55	5.48	1.82	−41.85 ^c^	0.67
	Vertical insertion	−6.60 ^a^	8.25	4.95	1.61	−25.43 ^c^	0.64
50% ETc	Horizontal insertion	4.33 ^a^	4.43	0.98	1.10	1.52	0.96
	Laid horizontal	−13.50 ^a^	14.10	4.08	1.62	−31.47 ^c^	0.80
	Vertical insertion	2.84	3.25	1.57	1.24	−4.09	0.94
	**Campbell Scientific 655**						
100% ETc (n = 23)	Horizontal insertion	10.77 ^a^	10.94	1.94	1.33	0.31	0.84
	Laid horizontal	3.13	6.92	6.17	2.44	−42.99 ^c^	0.72
	Vertical insertion	0.35	8.62	8.61	3.71	−86.94 ^c^	0.92
75% ETc (n = 23)	Horizontal insertion	3.88 ^a^	4.95	3.08	1.28	−4.57	0.72
	Laid horizontal	−14.77 ^a^	16.85	8.11	2.09	−48.14 ^c^	0.53
	Vertical insertion	−15.99 ^a^	17.11	6.09	1.87	−42.60 ^c^	0.63
50% ETc (n = 17)	Horizontal insertion	8.32 ^a^	8.40	1.06	0.72	15.86 ^c^	0.85
	Laid horizontal	−11.69 ^a^	11.75	1.25	0.66	−2.35	0.79
	Vertical insertion	−0.37	0.93	0.86	1.43	−11.70 ^c^	0.92
	**Watermark 200SS**						
100% ETc (n = 23)	Horizontal insertion	−7.06 ^a^	7.38	2.16	1.19	−13.02 ^c^	0.74
	Laid horizontal	−7.85 ^a^	8.36	2.88	1.33	−18.31 ^c^	0.67
	Vertical insertion	−8.29 ^a^	9.13	3.84	1.41	−21.36 ^c^	0.56
75% ETc (n = 23)	Horizontal insertion	−9.79 ^a^	10.01	2.09	0.77	−2.60	0.68
	Laid horizontal	−10.12 ^a^	10.33	2.10	0.85	−5.61	0.70
	Vertical insertion	−10.32 ^a^	10.52	2.03	0.84	−5.43	0.71
50% ETc (n = 17)	Horizontal insertion	−9.54 ^a^	9.68	1.64	0.41	6.67 ^c^	0.77
	Laid horizontal	–9.91 ^a^	10.10	1.94	0.27	9.96 ^c^	0.71
	Vertical insertion	−9.81 ^a^	9.97	1.77	0.36	7.57 ^c^	0.70

^a^ MD (mean difference) is significantly different from zero at *p* < 0.05 level. ^b^ Slope was significantly different from zero at *p* < 0.05 level. ^c^ Intercept is significantly different from zero at *p* < 0.05 level. RMSE indicates root mean square error. SD indicates standard deviation.

**Table 5:** Field correction equations developed for four soil water sensors in the Pullman clay loam soil at Bushland, TX, USA.

Field Correction	Equation Type	Equation	MD (%)	RMSE (%)	*R* ^2^
Acclima horizontal insertion (n = 69)	Linear	θ_v_ = 0.56 × θ_vi_ + 12.69	−0.00007	1.90	0.75
	Exponential	θ_v_ = 16.31 × $e^{0.019\times \theta_{\mathrm{vi}}}$	−0.065	1.94	0.73
	Logarithmic	θ_v_ = 16.12 × ln (θ_vi_) − 25.15	0.0017	1.91	0.75
	Quadratic	θ_v_ = −0.0062 × (θ_vi_)^2^ + 0.93 × θ_vi_ + 7.31	0.0026	1.89	0.75
Decagon horizontal insertion (n = 69)	Linear	θ_v_ = 0.49 × θ_vi_ + 14.02	−0.00015	2.47	0.57
	Exponential	θ_v_ = 17.19 × $e^{0.017\times \theta_{\mathrm{vi}}}$	−0.074	2.44	0.55
	Logarithmic	θ_v_ = 14.12 × ln (θ_vi_) − 18.91	−0.00048	2.58	0.53
	Quadratic	θ_v_ = 0.016 × (θ_vi_)^2^ − 0.46 × θ_vi_ + 28.22	0.030	2.40	0.60
Decagon vertical insertion (n = 69)	Linear	θ_v_ = 0.56 × θ_vi_ + 11.71	0.0011	1.78	0.78
	Exponential	θ_v_ = 15.76 × $e^{0.019\times \theta_{\mathrm{vi}}}$	−0.063	1.82	0.76
	Logarithmic	θ_v_ = 17.58 × ln (θ_vi_) − 31.02	−0.00048	1.73	0.79
	Quadratic	θ_v_ = −0.018 × (θ_vi_)^2^ + 1.72 × θ_vi_ − 6.19	−0.048	1.72	0.79
Campbell Scientific horizontal insertion (n = 63)	Linear	θ_v_ = 0.55 × θ_vi_ + 8.84	0.0010	2.18	0.67
	Exponential	θ_v_ = 14.27 × $e^{0.019\times \theta_{\mathrm{vi}}}$	−0.14	2.19	0.66
	Logarithmic	θ_v_ = 20.11 × ln (θ_vi_) − 43.33	0.0015	2.19	0.67
	Quadratic	θ_v_ = −0.0001 × (θ_vi_)^2^ + 0.55 × θ_vi_ + 8.69	0.0072	2.18	0.67
Campbell Scientific vertical insertion (n = 63)	Linear	θ_v_ = 0.35 × θ_vi_ + 19.14	0.00048	2.02	0.72
	Exponential	θ_v_ = 20.45 × $e^{0.012\times \theta_{\mathrm{vi}}}$	−0.088	2.09	0.69
	Logarithmic	θ_v_ = 10.40 × ln (θ_vi_) − 5.30	−0.00060	1.90	0.75
	Quadratic	θ_v_ = −0.011 × (θ_vi_)^2^ + 1.04 × θ_vi_ + 9.25	0.035	1.83	0.77
Watermark horizontal insertion (n = 63)	Linear	θ_v_ = 0.78 × θ_vi_ + 12.33	−0.00005	2.04	0.71
	Exponential	θ_v_ = 16.16 × $e^{0.027\times \theta_{\mathrm{vi}}}$	−0.066	2.18	0.69
	Logarithmic	θ_v_ = 18.14 × ln (θ_vi_) − 26.18	−0.00004	1.87	0.76
	Quadratic	θ_v_ = −0.057 × (θ_vi_)^2^ + 3.41 × θ_vi_ − 17.03	−0.0039	1.70	0.80
Watermark laid horizontal orientation (n = 63)	Linear	θ_v_ = 0.80 × θ_vi_ + 12.11	−0.00051	2.03	0.71
	Exponential	θ_v_ = 16.04 × $e^{0.028\times \theta_{\mathrm{vi}}}$	−0.071	2.28	0.69
	Logarithmic	θ_v_ = 19.07 × ln (θ_vi_) − 28.81	0.0016	1.77	0.78
	Quadratic	θ_v_ = −0.054 × (θ_vi_)^2^ + 3.37 × θ_vi_ − 17.14	−0.014	1.46	0.85
Watermark vertical insertion (n = 63)	Linear	θ_v_ = 0.73 × θ_vi_ + 13.75	−0.0011	2.21	0.66
	Exponential	θ_v_ = 17.01 × $e^{0.025\times \theta_{\mathrm{vi}}}$	−0.10	2.42	0.63
	Logarithmic	θ_v_ = 17.64 × ln (θ_vi_) − 24.3	0.00054	1.96	0.74
	Quadratic	θ_v_ = −0.051 × (θ_vi_)^2^ + 3.19 × θ_vi_ − 14.39	−0.012	1.66	0.81
Proposed evaluation criteria			±1.0	<3.5	>0.65

θ_vi_ is factory calibrated θ_v_ as an input in percent (%) and not directly as a fraction (m^3^ m^−3^). MD indicates mean difference. RMSE indicates root mean square error.

## Supplementary Materials

The following is available online at https://www.mdpi.com/1424-8220/19/13/2872/s1, Table S1: Soil information for Pullman clay loam soil at Bushland, TX, USA, Figure S1: Illustration of the multiple sensor installation orientations including laid horizontal, vertical insertion, and horizontal insertion at the study site. Note the extensive amount of wiring leads in the trench with the sensor array., Figure S2: Graphical and statistical comparisons of sensor and neutron moisture meter-derived soil profile water storage values in the upper 0.9 m soil profile under the 100% crop evapotranspiration treatment. MD indicates mean difference; RMSE indicates root mean square error; * indicates a significant difference at *p* < 0.05; ** indicates a significant difference at *p* < 0.01., Descriptions of Neutron Moisture Meter and Soil Water Sensors.
