# Supplementary material for: Soil Water Sensor Performance and Corrections with Multiple Installation Orientations and Depths under Three Agricultural Irrigation Treatments
Source: Sensors (Basel). 2019 Jun 28;19(13):2872. doi: 10.3390/s19132872 (PMC6651115; doi:10.3390/s19132872)
Supplement: Supplementary file 1 [file sensors-19-02872-s001.pdf]

Supplementary Information

Soil Water Sensor Performance and Corrections with Multiple Installation Orientations and Depths under Three Agricultural Irrigation Treatments

Yong Chen [1,*], Gary W. Marek [2], Thomas H. Marek [3], Kevin R. Heflin [3], Dana O. Porter [4], Jerry E. Moorhead [2] and David K. Brauer [2]

[1] Department of Ecosystem Science and Management, Texas A&M University, College Station, TX 77843, USA
[2] USDA-ARS Conservation and Production Research Laboratory, 300 Simmons Rd., Unit 10, Bushland, TX 79012, USA
[3] Texas A&M AgriLife Research and Extension Center at Amarillo, 6500 Amarillo Blvd. W., Amarillo, TX 79106, USA
[4] Texas A&M AgriLife Research and Extension Center at Lubbock, 1102 E FM 1294, Lubbock, TX 79403, USA
* Correspondence: yongchen@neo.tamu.edu

Table S1. Soil information for Pullman clay loam soil at Bushland, TX, USA.

Soil information	Layer 1	Layer 2	Layer 3	Layer 4
Depth (m)	0–0.18	0.18–0.74	0.74–1.35	1.35–2.29
Bulk density (g cm^{-3})	1.35	1.47	1.48	1.41
Plant available water capacity (mm H_2O per mm soil)	0.15	0.15	0.15	0.14
Saturated hydraulic conductivity (mm hr^{-1})	5.72	2.16	2.16	5.72
Clay content (% soil mass)	30.3	37.4	38.4	36.5
Silt content (% soil mass)	49.3	45.0	41.2	44.0
Sand content (% soil mass)	20.4	17.6	20.4	19.5

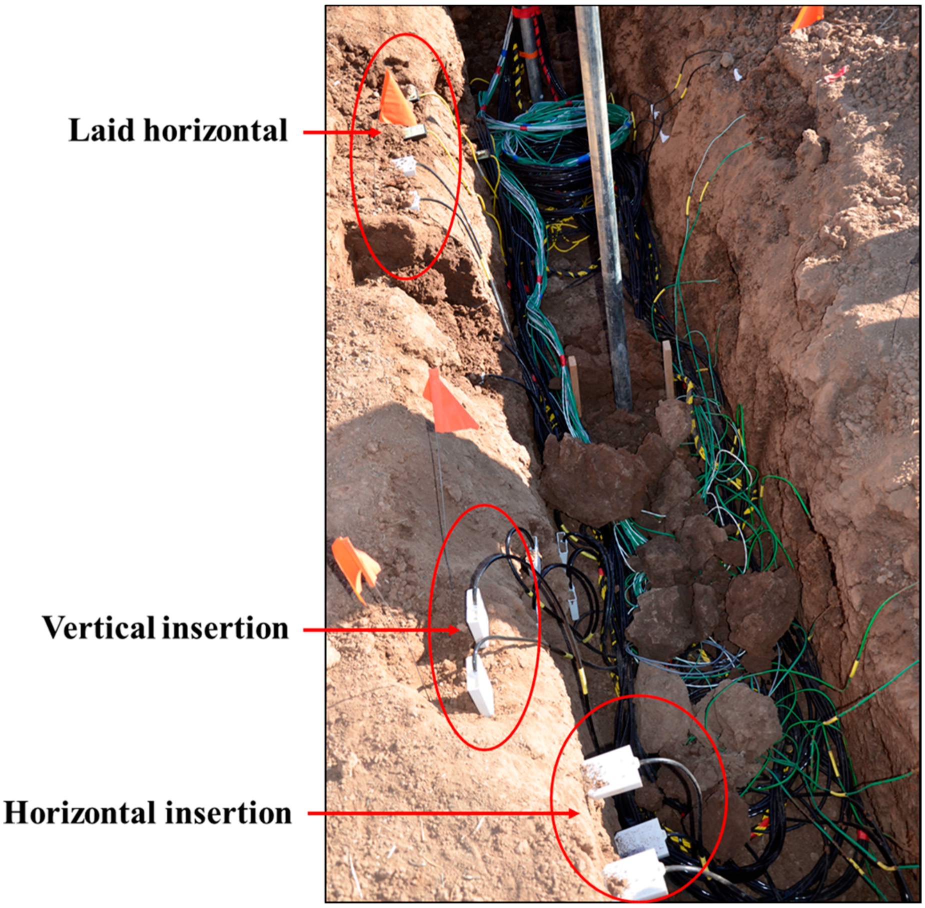

Figure S1. Illustration of the multiple sensor installation orientations including laid horizontal, vertical insertion, and horizontal insertion at the study site. Note the extensive amount of wiring leads in the trench with the sensor array.

Descriptions of Neutron Moisture Meter and Soil Water Sensors

1. Neutron Moisture Meter (NMM)

Campbell Pacific Nuclear (CPN) model 503DR NMMs were used in this study (InstroTek, Inc., Raleigh, NC, USA) to measure θ_v (m^3 m^{-3}). The meter consists of a radioactive source and detector. The source is lowered into the soil through an access tube, and measurements are recorded for desired depths as determined by stops installed on the source cable. Measurements at depths of 10, 30, 50, 70, 90, 110, 130, 150, 170, 190, 210, and 230 cm below the ground surface were recorded in this study although a maximum rooting depth of 150 cm was determined for corn. The use of a depth control stand served to control probe depth relative to the soil surface and allowed for successful use at shallow soil depths [1]. Fast neutrons are scattered into the surrounding soil and lose energy and slow after colliding with hydrogen nuclei. The detector counts the low energy “slow” neutrons, and the Neutron Count is compared to a Standard Count to estimate θ_v using a linear calibration equation with a slope of “a” and intercept of “b”.

$$\theta_v = a \times \left[\frac{\text{Neutron Count}}{\text{Standard Count}}\right] + b \quad (1)$$

The Standard Count is also used to check NMM performance and verifies that the detector is operating correctly. Typically, the NMM is not sensitive to variations of soil temperature and salinity [2]. However, it can be affected by soil clay content, organic matter content, texture, and chemical elements [3]. Therefore, a site-specific calibration with the thermogravimetric method is

recommended. The NMMs used in this study were previously field calibrated for the Pullman clay loam soil at Bushland using the thermogravimetric method for the soil horizons of Ap (0–10 cm), Bt (30–110 cm), and Btk (130–230 cm) with a 1.0% accuracy [4]. Following on-site calibrations, NMMs can be used to evaluate other soil water monitoring devices [5].

2. Acclima 315L (ACC-315L)

The Acclima 315L (Acclima, Inc., Meridian, ID, USA) is a Time Domain Reflectometer (TDR) sensor with three parallel rods serving as the waveguide. The sensor head contains the necessary electronics and firmware to generate an electromagnetic (EM) pulse and construct a waveform to calculate the travel time of the EM wave, which is used to estimate the soil apparent permittivity (Ka; unitless) [6]. The sensor also measures soil temperature. The ACC-315L measures apparent electrical conductivity (EC) according to the Giese and Tiemann method [7]. The sensor outputs θ_v (m^3 m^{-3} or %) from 0 to 100% with a resolution of ±0.1%, soil temperature from −40 to 60 °C with a resolution of ±0.1 °C, and soil EC from 0 to 5000 μS cm^{-1} with an accuracy dependent upon the reading range. The following equation is used to determine θ_v from Ka [8].

$$\theta_v = 4.3 \times 10^{-6} (Ka^3) - 5.5 \times 10^{-4} (Ka^2) + 2.92 \times 10^{-2} (Ka) - 5.3 \times 10^{-2} \quad (2)$$

3. Decagon GS1 (DEC-GS1)

The Decagon GS1 (METER Group, Inc., Pullman, WA, USA) is based on the capacitance and frequency domain techniques. It estimates θ_v by generating an EM pulse to measure the permittivity of the surrounding medium. The sensor provides oscillating waves to the sensor rods where the charge/discharge time is related to the dielectric permittivity of the material. The DEC-GS1 determines the charge and reads a raw value (RV; mV), which can then be transformed into θ_v (Equation 3). The sensor includes a two-rod design, with each rod measuring 5.5 cm in length. It operates at a frequency of 70 MHz with a measurement range indicated by the manufacturer of 0 to 57%. θ_v is estimated based on the following:

$$\theta_v = 3.62 \times 10^{-4}\, RV - 0.554 \quad (3)$$

4. Campbell Scientific 655 (CS-655)

The Campbell Scientific 655 (Campbell Scientific, Inc., Logan, UT, USA) has two parallel rods forming an open-ended transmission line. It measures the two-way travel time of an EM pulse to determine a period average. The CS-655 also measures EC which is subsequently used with the period average to estimate Ka and then calculates θ_v based on the equation of Topp et al. [8] (Equation 2) [9]. The CS-655 also measures soil temperature. The sensor can measure θ_v ranges from 5% to 50% with an accuracy of ±3%. Soil EC measurements vary from 0 to 8000 μS cm^{-1} with an accuracy of ± (5% of reading + 50 μS m^{-1}). Soil temperature measurement range is −10 to +70 °C with an accuracy of ±0.5 °C.

5. Watermark 200SS (WM-200SS)

The Watermark 200SS (Irrometer, Inc., Riverside, CA, USA) is an electrical resistance device. The resistant electrodes are embedded within a granular matrix, and an electrical current is applied to obtain a resistance value. The water content resistance (SWR) changes as the water in the granular matrix changes in response to soil water tension changes. This resistance is measured with a voltage divider circuit with known resistances and correlated to resistance in centibars (cb) or kilopascals (kPa). The sensor outputs soil tension over a range of 0 to 200 kPa. To compare WM-200SS sensor with other sensors, a fitting equation ($\theta_v = 38.14 \times SWR^{-0.14}$) adopted from Varble and Chávez [10] for

a loamy sand soil was applied to estimate θ_v from soil water resistance from the WM-200SS. It is acknowledged that there is uncertainty by using this fitting equation.

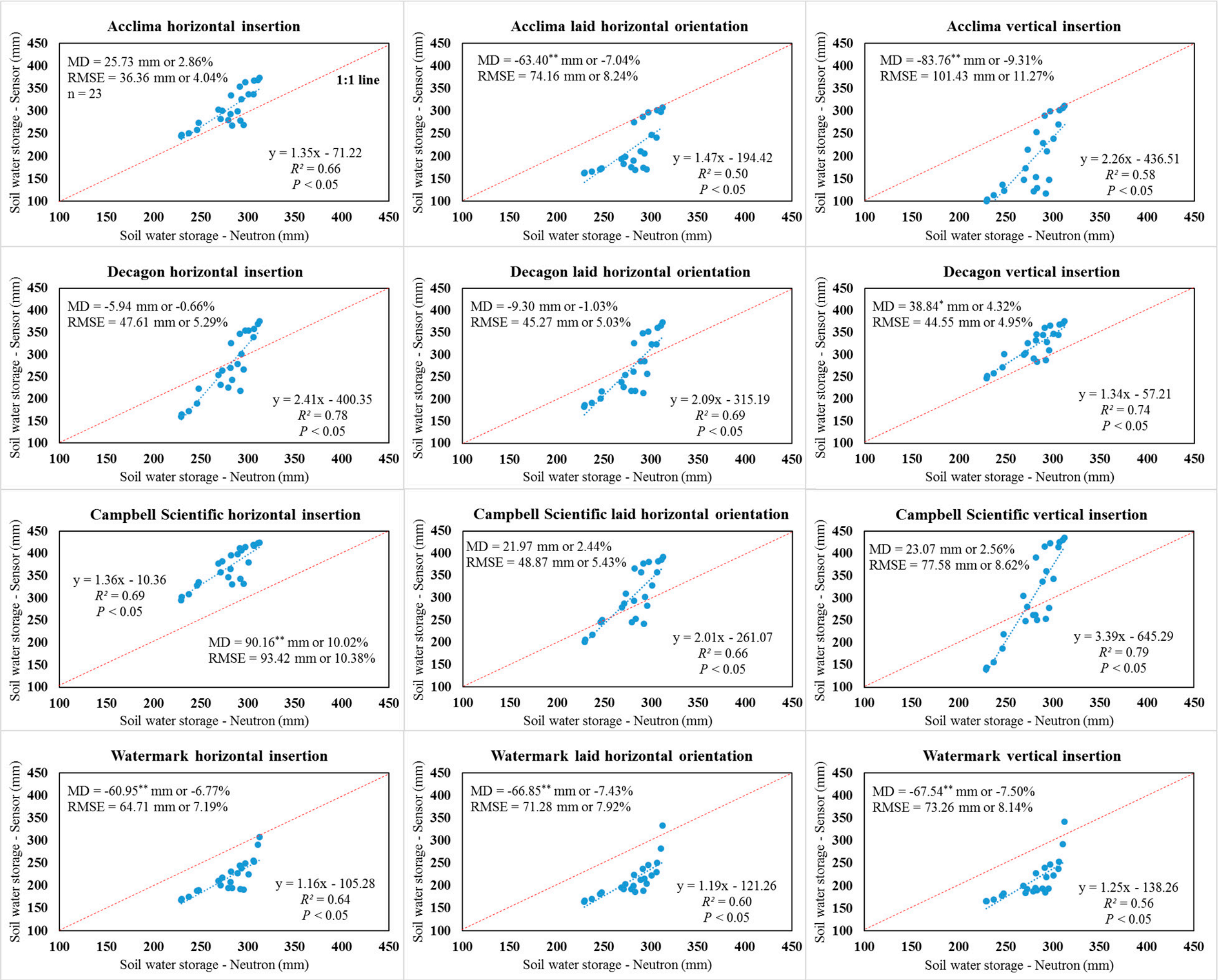

Figure S2. Graphical and statistical comparisons of sensor and neutron moisture meter-derived soil profile water storage values in the upper 0.9 m soil profile under the 100% crop evapotranspiration treatment. MD indicates mean difference; RMSE indicates root mean square error; * indicates a significant difference at $p < 0.05$; ** indicates a significant difference at $p < 0.01$.

References

1. Evett, S.R.; Tolk, J.A.; Howell, T.A. A depth control stand for improved accuracy with the neutron probe. *Vadose Zone J.* **2003**, *2*, 642–649.
2. Evett, S.R.; Tolk, J.A.; Howell T.A. Soil profile water content determination: sensor accuracy, axial response, calibration, temperature dependence, and precision. *Vadose Zone J.* **2006**; *5*, 894–907.
3. Hauser, V.L. Neutron meter calibration and error control. *Trans. ASAE* **1984**, *27*, 722–728.
4. Evett, S.R.; Steiner, J.L. Precision of neutron scattering and capacitance type soil water content gages from field calibration. *Soil Sci. Soc. Am. J.* **1995**, *59*, 961–968.
5. Leib, B.G.; Jabro, J.D.; Matthews, G.R. Field evaluation and performance comparison of soil moisture sensors. *Soil Sci.* **2003**, *168*, 396–408.
6. Schwartz, R.C.; Evett, S.R.; Anderson, S.K.; Anderson, D. Evaluation of a direct-coupled time domain reflectometer for determination of soil water content and bulk electrical conductivity. *Vadose Zone J.* **2016**, *15*, doi:10.2136/vzj2015.08.0115.
7. Giese, K.; Tiemann, R. Determination of the complex permittivity from thin-sample time domain reflectometry improved analysis of the step response waveform. *Adv. Mol. Relax. Processes* **1975**, *7*, 45–59.

8. Topp, G.C.; Davis, J.; Annan, A.P. Electromagnetic determination of soil water content: Measurements in coaxial transmission lines. *Water Resour. Res.* **1980**, *16*, 574–582.
9. Caldwell, T.G.; Bongiovanni, T.; Cosh, M.H.; Halley, C.; Young, M.H. Field and laboratory evaluation of the CS655 soil water content sensor. *Vadose Zone J.* **2018**, *17*, 170214.
10. Varble, J.L.; Chávez, J.L. Performance evaluation and calibration of soil water content and potential sensors for agricultural soils in eastern Colorado. *Agric. Water Manage.* **2011**, *101*, 93–106.
